# Non-invasive modulation of somatosensory evoked potentials by the application of static magnetic fields over the primary and supplementary motor cortices

**DOI:** 10.1038/srep34509

**Published:** 2016-10-04

**Authors:** Hikari Kirimoto, Akihiko Asao, Hiroyuki Tamaki, Hideaki Onishi

**Affiliations:** 1 Institute for Human Movement and Medical Sciences, Niigata University of Health and Welfare, Niigata, Japan

## Abstract

This study was performed to investigate the possibility of non-invasive modulation of SEPs by the application of transcranial static magnetic field stimulation (tSMS) over the primary motor cortex (M1) and supplementary motor cortex (SMA), and to measure the strength of the NdFeB magnetic field by using a gaussmeter. An NdFeB magnet or a non-magnetic stainless steel cylinder (for sham stimulation) was settled on the scalp over M1 and SMA of 14 subjects for periods of 15 min. SEPs following right median nerve stimulation were recorded before and immediately after, 5 min after, and 10 min after tSMS from sites C3′ and F3. Amplitudes of the N33 component of SEPs at C3′ significantly decreased immediately after tSMS over M1 by up to 20%. However, tSMS over the SMA did not affect the amplitude of any of the SEP components. At a distance of 2–3 cm (rough depth of the cortex), magnetic field strength was in the range of 110–190 mT. Our results that tSMS over M1 can reduce the amplitude of SEPs are consistent with those of low-frequency repeated TMS and cathodal tDCS studies. Therefore, tSMS could be a useful tool for modulating cortical somatosensory processing.

Olivielo *et al*.^1^ first described that the primary motor cortex (M1) in the human brain can be modulated by application of static magnetic fields (SMFs), as distinct from time-varying (electromagnetic) fields, through the scalp. They reported that 10 min of transcranial static magnetic field stimulation (tSMS) using a strongly powered cylindrical neodymium, iron, and boron (NdFeB) magnet can reduce the amplitude of motor evoked potentials (MEPs) for a few minutes after the magnet has been removed. In addition, they demonstrated that the polarity of the SMFs was not an important factor for this neuromodulation, and that tSMS was not directly associated with induced electric currents. Since then, tSMS received significant attention as a new non-invasive brain stimulation (NIBS) technique alongside conventional methods, such as repetitive transcranial magnetic stimulation (rTMS)^2^, theta-burst stimulation (TBS)^3^, repeated trains of four monophasic TMS pulses (quadripulse stimulation: QPS)^4^, and transcranial direct current stimulation (tDCS)^5^. Since the NdFeB magnet is an industrial product that is easily available, tSMS does not require expensive devices or high operational skill compared to other NIBS methods. Furthermore, conventional NIBS techniques have some adverse effects, such as itching, tingling, headache, and discomfort^6^, which are not observed with tSMS. An additional advantage is the inability of subjects to distinguish between the NdFeB magnet (for tSMS) and a non-magnetic stainless steel cylinder (for sham stimulation), allowing for conclusive controlled sham tSMS stimulation experiments and randomized controlled clinical trials.

In a recent study on tSMS application over M1, Silbert *et al*. showed that tSMS could reduce M1 excitability due to modulation of the resting motor threshold by TMS^7^, while Nojima *et al*. demonstrated enhanced short latency intracortical inhibition (SICI) by SMS^8^. In addition, we previously reported that SMFs over the sensorimotor cortex (C3 of the international 10–20 system of electrode placement) reduce the amplitudes of the N20 component of somatosensory evoked potentials (SEPs)^9^. Moreover, tSMS over the visual cortex increases alpha oscillations and slows visual search abilities^10^. These studies utilized the blocking and depressing effects of tSMS to create temporary cortical dysfunctions (‘virtual lesions’), which enabled functional examination of cortical regions.

In this study, we assessed whether tSMS with other magnet positions can modulate the amplitude of SEPs. The amplitude of the N20 component of SEPs recorded from the C3′ area (parietal component; 2.5 cm posterior to C3) is believed to be produced by a tangential generator located in Brodman’s area 3b of the primary somatosensory cortex (S1)^11^,^12^,^13^. While the generators of later components of SEPs (P25 and N33 from C3′) have not yet been definitively identified, several studies have suggested that M1 might be the generator of these later components. Previous studies reported that facilitatory rTMS^14^, TBS^15^, QPS^16^ and anodal tDCS over M1^17^ change the amplitude of the P25 and/or N33 component of SEPs at C3′. In our previous study, we demonstrated that tSMS over C3 changes the amplitude of the N20 component of SEPs at C3′; in that study, the NdFeB magnet seemed to cover S1 as well as M1, crossing over the central sulcus, and mainly or partly acted on S1. If tSMS over mainly M1 (TMS hot spot for hand muscles; 2–3 cm anterior to C3) could modulate the P25 and/or N33 component of SEP amplitudes at C3′ consistent with other NIBS studies, it would indicate that tSMS could be an alternative or even better tool for modulating cortical somatosensory processing, compared with conventional NIBS techniques which have some adverse effects. On the other hand, the supplementary motor area (SMA) is thought to be a generator of SEPs (N30 from F3)^18^,^19^. If these amplitudes of SEPs were affected by tSMS over SMA, it would demonstrate that tSMS is a beneficial technique for modulating the excitability of not only the sensorimotor and visual cortices, but also of the SMA, which is the motor association cortex. However, the effect of tSMS over SMA on SEP amplitudes has never been examined. We also aimed to determine how much the actual strength of the magnetic field is a function of the distance from the base of the magnet. A recent study confirmed that the magnetic flux density ranges between 120 and 200 mT at 2–3 cm from the magnet surface with high reproducibility^20^. This strength is sufficient to reach the majority of the cortical targets and alter biological functions^21^,^22^. However, we used a different type of NdFeB magnet. These magnets are industrial products produced by different manufacturers and for which information on the consistency of the magnetic strength throughout the magnet is still required. Therefore, we first assessed the strength of the magnetic field of the NdFeB magnet that was used in this study. Hence, the present study had two aims: to investigate the possibility of non-invasive modulation of SEPs by the application of tSMS over M1 and SMA in healthy humans, and to measure the strength of the NdFeB magnetic field using a gaussmeter.

## Results

### Amplitudes of SEPs

**Figure 1:**
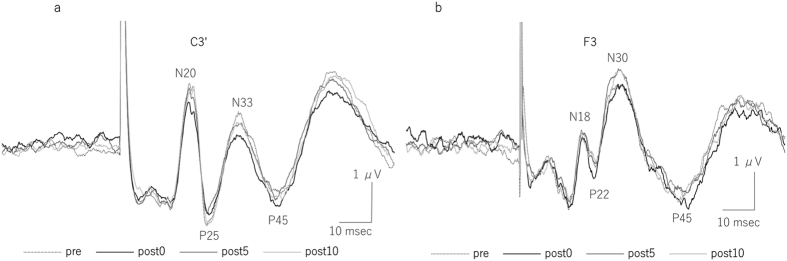
Grand averaged SEP waveforms recorded from C3′ (**a**) and F3 (**b**) after right median nerve stimulation immediately after tSMS over M1. Note the attenuation of the amplitude of the N33 component of SEPs at C3′ after tSMS over M1.

**Figure 2:**
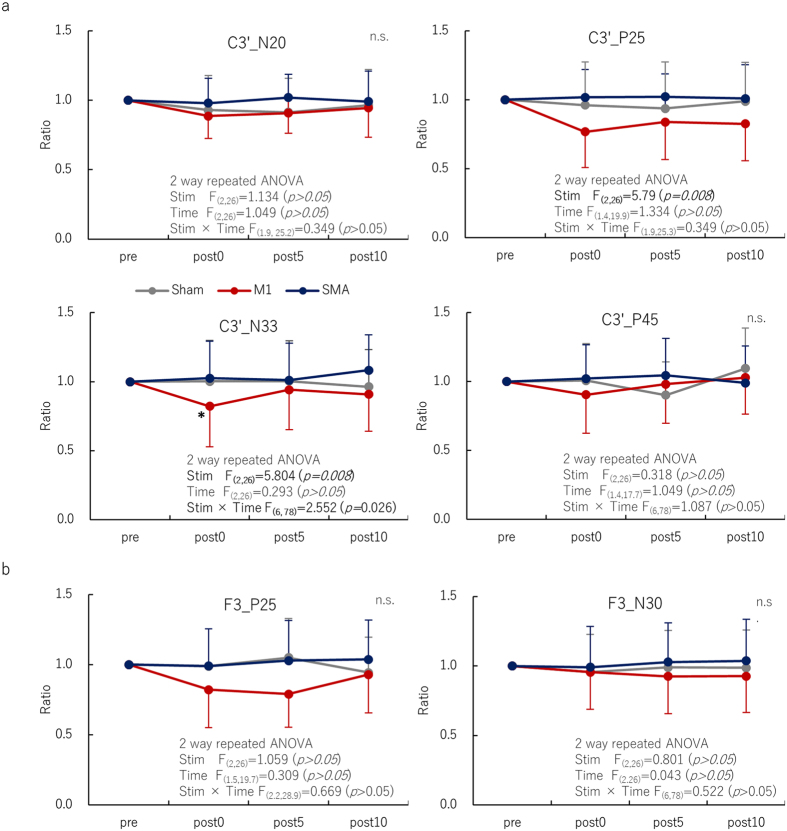
Serial changes in SEP amplitudes (N20, P25, N33, P45) from C3′ (**a**) and (P22, N30) from F3 (**b**) before, immediately after, 5 min after, and 10 min after tSMS over M1 and SMA for periods of 15 min and after sham stimulation. For the N33 (C3′) component of tSMS over M1, post hoc analysis showed a significant difference between sham stimulation and tSMS over the SMA immediately after the intervention (mean ± SEM) (*p < 0.05).

Figure 1 shows grand averaged waveforms of SEPs recorded before and immediately, 5 min, and 10 min after 15 min of tSMS over M1 from the C3′ (a) and F3 positions (b). The amplitudes of N33 of C3′ (parietal component) significantly decreased immediately after tSMS. The amplitudes of N33 (C3′) before tSMS in each stimulus condition were comparable: sham 2.94 ± 0.22 μV, tSMS over M1 3.56 ± 0.31 μV, and tSMS over SMA 3.07 ± 0.29 μV, respectively. For the P25 component of SEPs recorded from C3′, two-way repeated measures ANOVA revealed a significant main effect of the stimulus site of tSMS (*F*_2, 26_ = 5.79, *p* = 0.008), but no significant main effect of time and the interaction between the stimulus site of tSMS and time was observed. For the N33 component of SEPs recorded from C3′, two-way repeated measures ANOVA revealed a significant main effect of the stimulus site of tSMS (*F*_2, 26_ = 5.804, *p* = 0.008) and the interaction between the stimulus site of tSMS and time (*F*_2, 78_ = 2.552, *p* = 0.026), but no significant main effect of time was observed. Under the M1 stimulation condition, the amplitude of the N33 component of SEPs was significantly reduced immediately after 15 min of tSMS (82 ± 2.3% of baseline, *p* = 0.001) compared with sham tSMS and SMA stimulation conditions. This decrease in amplitude of the N33 component of SEPs under M1 stimulation conditions was observed, although not significantly, at 5 min (94 ± 2.9%) and 10 min (91 ± 5.8%) after tSMS. Under all stimulus conditions, there were no remarkable effects on the amplitudes of the other SEP components (N20 and P45 from C3′ and P25 and N30 from F3) (Fig. 2a,b).

### Strength of the NdFeB magnetic field

**Figure 3:**
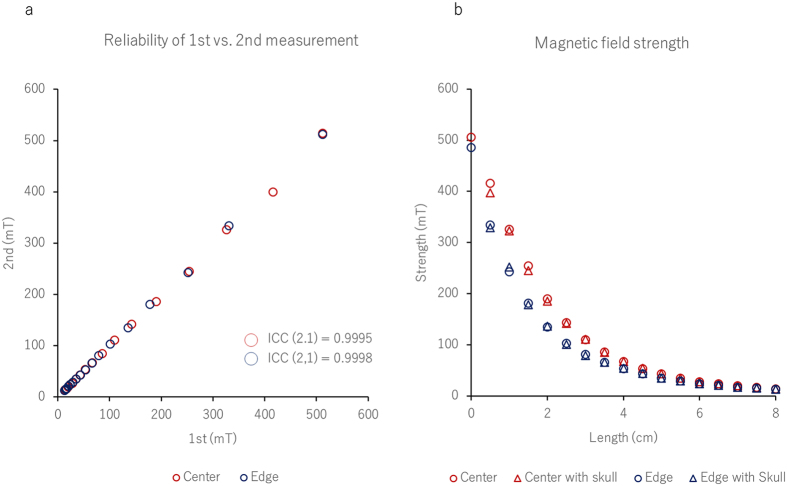
Relationship between the first and second measurements of magnetic field strength (**a**). High reproducibility at both the center and edge of the NdFeB magnet indicates the consistency of the field strength in this study. Relationship between magnetic field strength and distance from the surface of the NdFeB magnet (**b**). We confirmed that at 2–3 cm from the magnet surface, the magnetic field strength ranged between 110 and 190 mT, which is strong enough to reach most cortical targets and to obtain biological effects^21^,^22^, such as altering the function of the membrane ion channels, regardless of the presence or absence of the skull. Note the attenuation of reduction in magnetic flux density at the edge of the magnet by 30% of the value at the center.

Figure 3a shows the association between the first and second magnetic field strength measurements. Inter-measurement reproducibility from the center [ICC (2, 1) = 0.9995] and edge of the magnet [ICC (2, 1) = 0.9998] were both excellent. Figure 3b shows the association between the magnetic field strength and the distance from the surface of the NdFeB magnet. In accordance with Coulomb’s law, the magnetic field strength decreases in inverse proportion to the square of the distance. We confirmed that the magnetic field strength at the surface of the magnet was 512 mT, and the strength 2–3 cm from the surface of the center of the magnet ranged between 110 and 190 mT, regardless of the presence or absence of the skull. On the other hand, the magnetic field strength of the edge of the NdFeB magnet ranged between 80 and 140 mT at 2–3 cm from the magnet surface.

## Discussion

This study demonstrated that the amplitude of the N33 component of SEPs at C3′ decreased significantly immediately after a 15-min period of tSMS over M1 by up to 20%, returning to baseline by 5 min after the intervention. Our results suggest that the components of SEPs that are reduced depend on the tSMS stimulation site: for instance, tSMS over the sensorimotor cortex (C3) modulates only the N20 component of SEPs, as shown in our previous study^9^, while the amplitude of N33 is affected by tSMS over the M1. This result, showing that tSMS over M1 can reduce the amplitude of the N33 component of SEPs at C3′ is partly consistent with the results of rTMS^14^, TBS^15^, QPS^16^, and tDCS^17^,^23^ shown in previous M1 studies. Therefore, tSMS could be a useful novel non-invasive brain stimulation (NIBS) tool for modulating cortical somatosensory processing.

Many cellular and animal studies have attempted to demonstrate alteration of the central nervous function by SMFs^22^,^24^,^25^,^26^,^27^,^28^,^29^,^30^ and to show that moderate-intensity SMFs could induce magnetic reorientation of membrane phospholipids due to diamagnetic anisotropy effects^21^,^31^,^32^. Of great interest to cerebral excitability are the results that SMFs are not associated with induced electric currents during activation, deactivation, or movement within the field^22^. Furthermore, they alter the activation threshold velocity of voltage-gated sodium channels^21^,^22^,^27^,^29^ and voltage-gated calcium channels^21^,^22^,^28^. Slow calcium influx and increased intracellular calcium ion stores caused by an impedance of calcium channels are thought to trigger long-term depression^33^,^34^. These results, together with those of previous cellular and animal studies^25^,^27^, indicate that SMFs applied to the human cortex act primarily at the synapse and alter membrane ion channels. However, it has also been postulated that tSMS reduces corticomotor excitability in association with modulation of the resting motor threshold, as with TMS^7^. Hence, tSMS may not only alter the function of membrane ion channels, but also reduce membrane excitability, suggesting a possible role for non-synaptic (intrinsic) plasticity mechanisms. In this study, we confirmed that the NdFeB magnet used produced a magnetic field that was sufficient to reach most cortical targets (at a strength ranging between 110 and 190 mT 2–3 cm from the magnet surface)^13^,^14^, and to produce biological effects by diamagnetic anisotropy effects, as stated above. This result is in concurrence with those of previous studies that the NdFeB magnet with a surface magnetic flux density of about 500 mT and a nominal strength of 765 N (78 kgf) produces a magnetic flux density ranging between 120 and 200 mT at a distance of 2–3 cm from the magnet surface^20^. It is important to note here that it may be critical for the center of the magnet to accurately superimpose the target area of the human cortex during tSMS, due to reduction in the magnetic flux density at the edge of the magnet by 30% of the value at the center.

Interestingly enough, the reduction in S1 excitability of up to 20% seen in this study is in accordance with those described in our recent study and other previous studies that demonstrated a reduction in motor cortex excitability of up to 20–25%, as revealed by TMS, by applying tSMS over M1 for 10 or 15 min^1^,^7^,^8^. We infer that the inhibitory modulation of human cortical excitability, due to diamagnetic anisotropy effects of tSMS, might be induced equally in both primary motor and somatosensory cortices. It is possible that purposeful modulation of the direction and magnitude of NIBS-induced plasticity is determined by gender, age, time of day, and pharmacotherapy^35^. Further study will be required to clarify whether or not these determinants alter the effects of tSMS.

Previous studies reported that facilitatory rTMS^14^, TBS^15^, and QPS^16^ over M1 increase the amplitude of the N33 component of SEPs at C3′, and anodal tDCS over M1 increases the amplitudes of the P25 and N33 components of SEPs at C3′^17^. In addition, our previous magnetoencephalography (MEG) study demonstrated that the source strengths of the P35m and P60m components of somatosensory evoked magnetic fields (SEFs) increased after anodal tDCS was applied over M1^23^. Further, none of these facilitatory NIBS techniques altered the N20 component of SEPs and SEFs. While tSMS has an inhibitory effect, the affected components are consistent with those reported in other studies. However, we cannot explain for certain why only the N33 component of SEPs at C3′ decreased after tSMS over the M1, while the amplitudes of other SEP components (N20 and P45 from C3′ and P25 and N30 from F3) indicated no remarkable effects under both M1 and SMA stimulation conditions. N20 at C3′ is the earliest localized scalp potential and is believed to be produced by a tangential generator located in Brodman’s area 3b of the somatosensory cortex^11^,^12^,^13^. Meanwhile, there are many views on the generation of later components, such as N33 at C3′ from areas 1, 2^18^, and 4^36^ and N30 at F3 from area 4 and/or 6^3^,^37^,^38^, although these have not yet been definitively identified. In this study, the area stimulated by SMFs, the hot-spot of the abductor pollicis brevis muscle, was approximately 2–3 cm anterior to C3. Considering the 5 cm diameter of the NdFeB magnet, and reduction in the magnetic flux density at the edge of the magnet by 30% of the value at the center, tSMS over M1 in this study might have acted mainly on the pre- rather than the post-central sulcus. The hypothesis that the generator of N20 is area 3b, and that of N33 is area 4, can be inferred from the fact that tSMS over the sensorimotor cortex changes N20, as seen in our previous study^9^, and N33 changed after tSMS was applied over M1 in this study. Meanwhile, we showed that tSMS over the SMA did not influence the amplitude of any of the SEP components. The SMA is likely to be a more difficult area to target with tSMS than M1, since it is located in the interhemispheric fissure, which would result in the magnetic field strength of tSMS being attenuated to non-effective levels. In conventional NIBS studies, facilitatory or inhibitory stimulation over M1 or S1 resulted in concomitant reduction in pain perception and the amplitude of evoked potentials induced by laser stimulation^39^. Therefore, there is room for further investigation as to whether tSMS can reduce pain perception and the amplitude of pain evoked potentials.

In conclusion, we revealed that tSMS over M1 can induce plastic changes in primary sensorimotor areas and confirmed the consistency of the strength of the NdFeB magnetic field in altering the excitability of cortical function.

## Methods

### Subjects

Fourteen healthy subjects (9 males and 5 females, 21–37 years old) participated in this study. None of them were receiving medical treatment for any condition. Informed consent was obtained before beginning the experiment, which was conducted according to the Declaration of Helsinki. The experimental procedures were approved by the Ethics Committee of the Niigata University of Health and Welfare. Based on administration of the Oldfield inventory^40^, the handedness scores of all subjects ranged from 0.9 to 1.0 (strongly right-handed).

### Sample size calculation

The sample size of this study was calculated using the following formula:


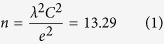


where *λ* is the estimated non-centrality parameter, which is 1.96 for the 95% confidence interval, *C* is the coefficient of variance of the SEP amplitudes in reference to the data in our previous study^9^, which is approximately 0.093, and *e* is the acceptable error rate of 0.05.

### Experimental procedure

**Figure 4:**
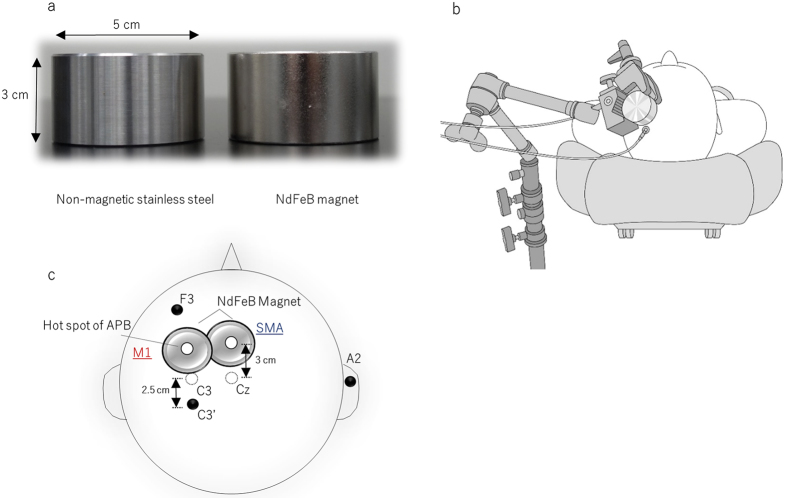
Photograph of a cylindrical neodymium (NdFeB) magnet (right) and non-magnetic stainless steel cylinder (left) (**a**). The NdFeB magnet (diameter, 50 mm; height, 30 mm) with a maximum energy density of 49 MGOe and a nominal strength of 862 N was used for tSMS, and the non-magnetic stainless steel cylinder of the same size, weight, and appearance was used for sham stimulation. Experimental setup: For tSMS, the NdFeB magnet and non-magnetic stainless steel cylinder were settled on the scalp using a movable arm-type lightning stand (**b**). The NdFeB magnet was centered over the representational field of the right abductor pollicis brevis (APB) muscle, as determined by a single-pulse TMS for M1 stimulation. For the SMA stimulation, the NdFeB magnet was centered 3 cm anterior to the Cz area of the international 10–20 system for electrode placement^41^. SEPs were recorded from the C3′ (parietal component; 2.5 cm posterior to C3) and F3 areas (frontal component) (**c**).

During the experiment, subjects were seated on a comfortable reclining armchair with a mounted headrest. For tSMS, a cylindrical neodymium magnet (NdFeB; diameter, 50 mm; height, 30 mm) with a surface magnetic flux density of 534 mT, a maximum energy density of 49 MGOe, and a strength of 862 N (88 kgf) nominal value was used (NeoMag Co., Ltd., Ichikawa, Japan). A non-magnetic stainless steel cylinder of the same size, weight, and appearance was used for sham stimulation (Fig. 4a). The NdFeB magnet or the non-magnetic stainless steel cylinder was settled on the scalp using a movable arm-type lightning stand (C-stand, Avenger, Cassola, Italy) (Fig. 4b). For M1 stimulation, the NdFeB magnet was centered over the representational field of the right abductor pollicis brevis muscle, as determined by a single-pulse TMS for M1 stimulation. For the SMA stimulation, the NdFeB magnet was centered 3 cm anterior to the Cz area of the international 10–20 system for electrode placement^41^ (Fig. 4c). Somatosensory evoked potentials (SEPs) following right median nerve stimulation were recorded before and immediately, 5 min, and 10 min after tSMS for 15 min. Based on the accepted method for attaching scalp electrodes for experiments or EEG tests, SEPs were recorded from the F3 (frontal component) and C3′ (parietal component; 2.5 cm posterior to C3) positions of the international 10–20 system using silver-silver chloride electrodes (1.0 cm diameter). A reference electrode was placed on the right earlobe (A2). Since Oliviero *et al*.^1^ revealed that the effects of tSMS are not polarity dependent, we selected only south polarity for each session. SEPs were amplified with bandpass filters set at 1–3,000 Hz and the average of 300 responses was obtained. Brief electrical stimulation (0.2 ms) was delivered to the right median nerve at a frequency of 3.3 Hz (Nicolet Viking Quest EMG Machine, California, USA). The stimulus intensity was fixed at about 1.2 times the motor threshold. Sham stimulation with a non-magnetic stainless steel cylinder was applied over M1 for half the subjects and over the SMA for the remaining half. The use of two investigators allowed for double-blinding to be performed as follows. Investigator 1 performed the intervention that selected and placed the real magnet or sham stainless steel cylinder. Investigator 2, who was blinded to the type of intervention being performed, recorded SEPs and analyzed their amplitudes. To avoid carryover effects, all subjects participated in three experimental sessions (tSMS over M1 and SMA and sham stimulation over M1 or SMA) on separate days that were at least three days apart and in a counter-balanced order.

### Measurement of magnetic field strength

The strength of the NdFeB magnetic field was measured at intervals of 0.5 cm (Z-axis) from the magnet surface to a depth of 8 cm, both through the human skull and directly. We performed duplicate measurements at both the center and edge of the magnet surface, twice each. The time interval between the first and second measurements was 30 min. Using a Gaussmeter (5180, FW BELL, Orlando, USA), magnetic flux density was measured with an analog output at a 100 kHz sampling rate, ±0.75% accuracy and DC-30 kHz bandwidth. Signals from each measurement were confirmed using an oscilloscope and subsequently digitized at a sampling frequency of 100 kHz using a 16-bit A/D converter (Power Lab; ADInstruments, New South Wales, Australia) and stored on a personal computer for later analysis. Each instrument was calibrated immediately before data collection^42^.

### Data and statistical analysis

Peak-to-peak amplitudes of the four cortical SEP components (N20, P25, N35, and P45 for C3′; P22 and N30 for F3) were analyzed. The amplitude of each component was measured from the preceding peaks. Amplitudes of SEPs were normalized to those recorded before tSMS. These individual mean ratios were then averaged to give a grand mean ratio^43^. All data were expressed as mean ± SEM and were statistically analyzed by two-way repeated measures analysis of variance (ANOVA) with the stimulus site of tSMS (M1 vs. SMA vs. Sham) and time (before vs. immediately after vs. 5 min after vs. 10 min after tSMS). The sphericity of the data was tested by Mauchly’s test, and Greenhouse–Geisser corrected significance values were used when sphericity was lacking. Post hoc analysis was performed with Bonferroni’s correction for multiple comparisons. A difference was accepted as being significant at p < 0.05 for all analyses. A single measure of the interclass correlation coefficient, ICC (2, 1), was used to measure the reproducibility of measurement of magnetic field strength for the first and second measurements. Statistical analyses were performed using software (SPSS Statistical Package, ver. 21.0; IBM SPSS).

## Additional Information

**How to cite this article**: Kirimoto, H. *et al*. Non-invasive modulation of somatosensory evoked potentials by the application of static magnetic fields over the primary and supplementary motor cortices. *Sci. Rep.* **6**, 34509; doi: 10.1038/srep34509 (2016).
